# An Unexpected Finding During Oesophago-Gastro-Duodenoscopy in a Patient Presenting With Food Bolus Obstruction

**DOI:** 10.7759/cureus.50617

**Published:** 2023-12-16

**Authors:** Ali Elmdaah, Francesca Moroni

**Affiliations:** 1 Gastroenterology, Aberdeen Royal Infirmary, Aberdeen, GBR

**Keywords:** oesophageal, rare cause of dysphagia, pseudodiverticulosis, food bolus, endosocpy

## Abstract

Oesophageal intraluminal pseudodiverticulosis is a rare benign condition of the oesophageal wall, with not many cases reported in the literature. Usually, patients present with dysphagia and food impaction in association with a proximal oesophageal stricture. Pathogenesis of the disease is not yet established; hence, it remains important to raise awareness about this distinctive pathology. Here, we present a case of a 62-year-old male admitted to Aberdeen Royal Infirmary, Scotland, UK, with a history of food bolus. Upper gastrointestinal endoscopy revealed food bolus impaction with underlying oesophageal pseudodiverticulosis in the distal two-thirds of the oesophagus.

## Introduction

Oesophageal intraluminal pseudodiverticulosis is a rare benign condition of the oesophageal wall characterised by tiny flask-shaped outpouching lesions [1,2]. The most common presenting symptom is dysphagia that is often accompanied by oesophageal strictures. Chronic alcoholism, diabetes mellitus and gastro-oesophageal reflux disease have been reported as associated comorbidities. The condition was first described by Mendl et al. in 1960 [3]. The pathophysiology of the disease is still unclear. Bender and Haddad suggested that diverticula formation might result from dysmotility associated with oesophagitis [4]. The diagnosis requires endoscopy and/or radiological imaging tests such as a computed tomography scan or barium swallow.

## Case presentation

A 62-year-old male presented to the emergency department 14 hours after food bolus impaction of a piece of beef steak. His main symptom was complete dysphagia. He reported no significant past medical history besides high blood pressure and gastro-oesophageal reflux disease. He had a 20 pack-year smoking history and a prior history of alcohol consumption. He had presented twice in the past with food bolus obstruction requiring endoscopic removal in the past three years.

**Figure 1:**
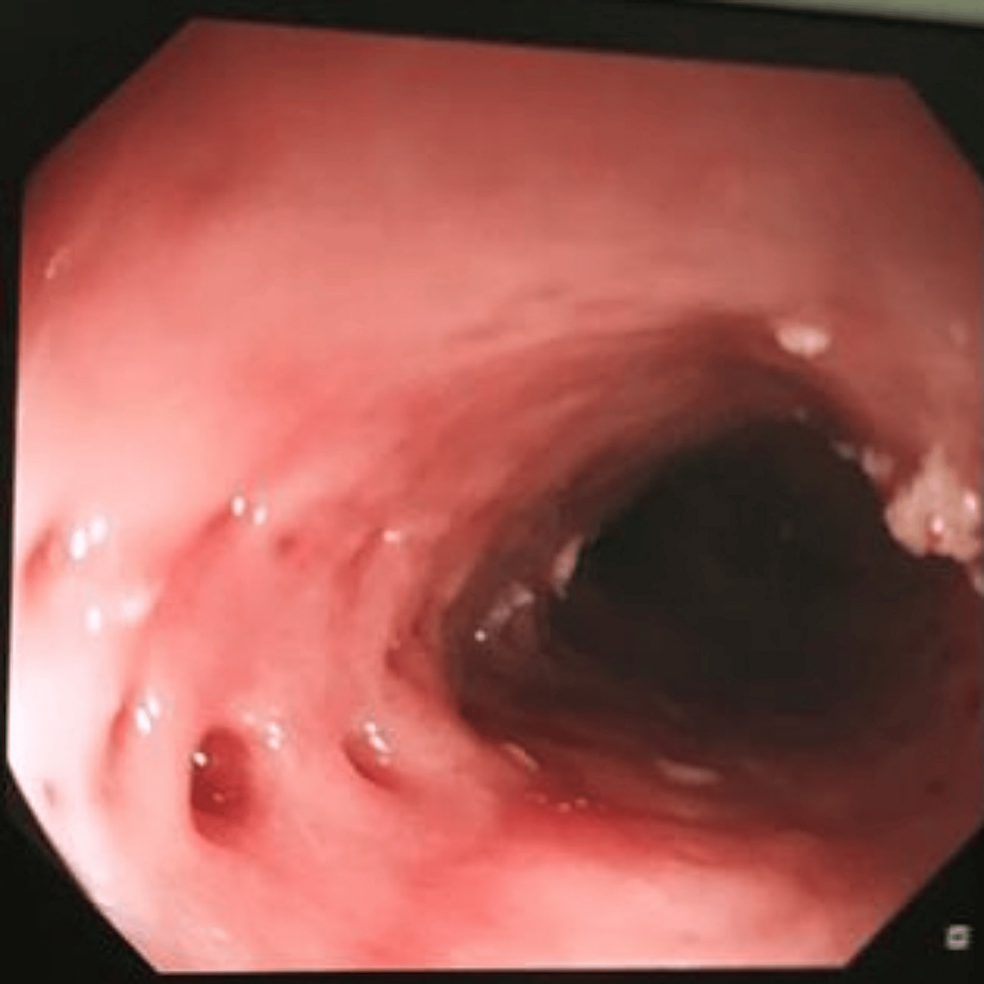
Intraluminal oesophageal pseudodiverticulosis

At presentation, he was hemodynamically stable; physical examination was unremarkable. Initial laboratory investigations showed a normal haemoglobin level, eosinophil count and C-reactive protein level. The patient was kept nil by mouth, and initial management included intravenous fluids, and IV hyoscine butylbromide, which unfortunately failed to resolve the impaction. The gastroenterology team was consulted and upper gastrointestinal (GI) endoscopy was arranged on the same day. The initial departmental endoscopy under conscious sedation revealed food bolus impaction at 20 cm from incisors. Different retrieval methods including foreign body net, rat tooth forceps and snare were not successful. CT of the thorax with contrast excluded oesophageal perforation. Endoscopy under general anesthesia was arranged to allow for more time to the procedure. This resulted in successful food bolus resolution. An unusual pathology was identified below the food bolus impaction area. Tiny flask-like outpouching lesions were noted in the middle and lower oesophagus with the appearance of pseudodiverticulosis seen (Figure 1) [5]. The patient was already on proton pump inhibitors and was discharged home next day with no complications. A follow-up endoscopy is planned to review the area of bolus impaction to exclude early stricture formation.

## Discussion

Oesophageal intramural pseudodiverticulos is mainly reported in men in the fifth and sixth decades of life [6]. Although the pathogenesis of the disease is not yet established, it is believed that chronic inflammation of the oesophagus due to gastroesophageal reflux disease may lead to the obstruction of the excretory glands and fibrosis of the submucosa [7]. It can be associated with diabetes mellitus, oesophageal dysmotilities, and chronic harmful alcohol use. Dysphagia is the most common presenting symptom in patients with oesophageal intraluminal pseudodiverticulosis. Only 20% of patients have diagnosis confirmed at endoscopy. Barium swallow radiography is a more sensitive diagnostic method. Imaging studies such as computed tomography could show diffuse oesophageal thickening. The most common complication of this condition is oesophageal stricture, accounting for 80%-90% of the reported cases [8]. Treatment involves treating the underlying condition such as gastroesophageal reflux disease and alcohol withdrawal. Endoscopic dilation improves symptoms of patients with oesophageal strictures [9].

## Conclusions

The management of oesophageal intraluminal pseudodiverticulosis is dependent on the patient’s symptoms. Around 10% of patients do not require treatment. The use of proton pump inhibitors can relieve symptoms of reflux oesophagitis. Oesophageal stricture is a common complication of pseudodiverticulosis and dilation may be required. Interestingly, in this case, the patient presented with a history of food bolus impaction and we did not identify oesophageal stricture at endoscopy.
